# Supplementary material for: Assessment of the Safety and Probiotic Properties of Enterococcus faecium B13 Isolated from Fermented Chili
Source: Microorganisms. 2024 May 15;12(5):994. doi: 10.3390/microorganisms12050994 (PMC11123876; doi:10.3390/microorganisms12050994)
Supplement: Supplementary file 1 [file microorganisms-12-00994-s001.zip › Table S2.pdf]

Table S2. Effect of *E. faecium* B13 on blood routine in mice.

blood indices	Control	B13	Reference range	*P*-value
White Blood Cell（$\times 10^9$/L）	2.13±1.15	1.7±0.46	0.80-6.80	0.428
Lymphocyte（$\times 10^9$/L）	1.62±1.36	1.24±0.52	0.70-5.70	0.553
Monocyte（$\times 10^9$/L）	0.15±0.06	0.1±0.00	0.00-0.30	0.182
Neutrophil（$\times 10^9$/L）	0.42±0.31	0.42±0.23	0.10-1.80	0.984
Red Blood Cell（$\times 10^{12}$/L）	8.49±0.39	8.49±0.40	6.36-9.42	0.989
Hemoglobin（g/L）	173.67±6.92	171.40±8.17	110.00-143.00	0.630
Blood Platelet（$\times 10^9$/L）	972.00±222.99	794.40±486.74	450-1590	0.442
