# Supplementary material for: Assessment of the Safety and Probiotic Properties of Enterococcus faecium B13 Isolated from Fermented Chili
Source: Microorganisms. 2024 May 15;12(5):994. doi: 10.3390/microorganisms12050994 (PMC11123876; doi:10.3390/microorganisms12050994)
Supplement: Supplementary file 1 [file microorganisms-12-00994-s001.zip › Table S3.pdf]

Table S3. Effects of *E. faecium* B13 on Organ Indexes in mice (100%).

Content	Control group	B13 group	*P* value
heart	0.57±0.1	0.53±0.1	0.399
liver	4.55±0.45	4.29±0.41	0.236
spleen	0.37±0.22	0.28±0.11	0.298
lungs	0.9±0.36	0.77±0.26	0.412
kidney	1.41±0.13	1.33±0.22	0.406
