# Supplementary material for: Assessment of the Safety and Probiotic Properties of Enterococcus faecium B13 Isolated from Fermented Chili
Source: Microorganisms. 2024 May 15;12(5):994. doi: 10.3390/microorganisms12050994 (PMC11123876; doi:10.3390/microorganisms12050994)
Supplement: Supplementary file 1 [file microorganisms-12-00994-s001.zip › Table S4.pdf]

Table S4. α-diversity indices.

Sample	ace	chao	shannon	simpson
B13	6391	6391	4.66828	0.034888
B13	5414	5414	4.558616	0.034936
B13	4104	4104	4.209894	0.060936
B13	5766	5766	4.479442	0.039732
B13	6331	6331	4.717356	0.034583
control	5716	5716	4.45398	0.036956
control	6244	6244	4.672001	0.035904
control	5934	5934	4.957037	0.027389
control	6552	6552	4.743967	0.032819
control	5118	5118	4.628274	0.034009
P value	0.8345	0.8345	0.5925	0.5925
